# Outlook on a Worldwide Forest Transition

**DOI:** 10.1371/journal.pone.0075890

**Published:** 2013-10-09

**Authors:** Chris Pagnutti, Chris T. Bauch, Madhur Anand

**Affiliations:** 1 School of Environmental Sciences, University of Guelph, Guelph, Ontario, Canada; 2 Department of Mathematics and Statistics, University of Guelph, Guelph, Ontario, Canada; 3 Department of Ecology and Evolutionary Biology, Princeton University, Princeton, New Jersey, United States of America; Cirad, France

## Abstract

It is not clear whether a worldwide “forest transition” to net reforestation will ever occur, and the need to address the main driver–agriculture–is compelling. We present a mathematical model of land use dynamics based on the world food equation that explains historical trends in global land use on the millennial scale. The model predicts that a global forest transition only occurs under a small and very specific range of parameter values (and hence seems unlikely) but if it does occur, it would have to occur within the next 70 years. In our baseline scenario, global forest cover continues to decline until it stabilizes within the next two centuries at 22% of global land cover, and wild pasture at 1.4%. Under other scenarios the model predicts unanticipated dynamics wherein a forest transition may relapse, heralding a second era of deforestation; this brings into question national-level forest transitions observed in recent decades, and suggests we need to expand our lexicon of possibilities beyond the simple “forest transition/no forest transition” dichotomy. This research also underscores that the challenge of feeding a growing population while conserving natural habitat will likely continue for decades to come.

## Introduction

According to the Food and Agriculture Organization (FAO) of the United Nations, global forest cover was reduced by more than 70 Mha since 1990, an area larger than France and roughly 0.5% of the global land area. The main driver of deforestation is agricultural expansion [1], [2]. It currently takes about 0.8 ha of cropland and pasture and 0.06 ha of urban land per person per year to feed and shelter the global population [3], [4]. At this rate, if population stabilizes at around 10 billion, then agricultural and urban land will cover over 67% of the Earth’s land area. Since around 15% of the global land area is classified as arid, [5] there will be little land area remaining for other purposes such as forest and wild pasture conservation. Despite the apparent demise of the world’s forests, over the last two centuries many countries, particularly in the industrialized world, have experienced a forest transition; that is, a transition from declining to expanding forested area [6], [7].

In the classical formulation of the forest transition the dynamics are simple: deforestation proceeds until the onset of the forest transition, after which time forest cover increases and eventually stabilizes. Some alternate approaches have been proposed to give the theory the flexibility needed to account for some real-world scenarios [8]. To this end, the possibility of a time lag between the end of the deforestation period and the start of the reforestation period has been suggested. However, there has been little focus on possible alternative forest cover dynamics that may ensue *after* the onset of the forest transition. For example, under what conditions may a forest transition be followed by a subsequent period of deforestation? In such a case, the original forest transition may be regarded as spurious.

The situation wherein a forest transition is followed by a second period of deforestation has been documented in the case of France where two forest transitions are believed to have occurred. The first was due to a decline of the French population during the time of the Black Death. The second was due to agricultural intensification among other factors [9]. Here we are interested in forest transitions of the second type, which we regard to be more important because they occur despite increasing population.

Land use models (e.g. IMAGE and GLOBIOM) generally assume that local parcels of land can be in one of several states and thus imply spatial localization [10]. These models are often quite complicated, and may account for a wide variety of ecological, biochemical, economic and political factors. Most studies of forest transitions and forest decline focus on spatial scales at the national and sub-national levels, and on temporal scales of a few decades. There are some important notable exceptions that have focused on policies and drivers that could potentially either trigger or inhibit a global forest transition in the future. [6], [7], [9], [11] However, few data-driven mathematical models have been developed to predict the timing of the forest transition and the ultimate (global) scale of deforestation [6].

In contrast, the model we present here is interpreted at the global level (i.e., is not spatially explicit) and the transitions between abundances of various land types do not correspond directly to transitions from one land type to another within a given patch. This removes confounding factors related to international trade, and other spatial processes that affect land use [12]. The model is capable of capturing historical land-use dynamics, and sheds some light on potential non-classical forest transition scenarios. The model is primarily based on the world food equation, is parameterized almost exclusively by historical data, and makes relatively few assumptions. Despite this simplicity, the model is capable of capturing estimates of historical land use dynamics at the global level going back several centuries. This is, to our knowledge, the first dynamic mathematical model based on the world food equation [10].

## Model

### Model Description

**Figure 1:**
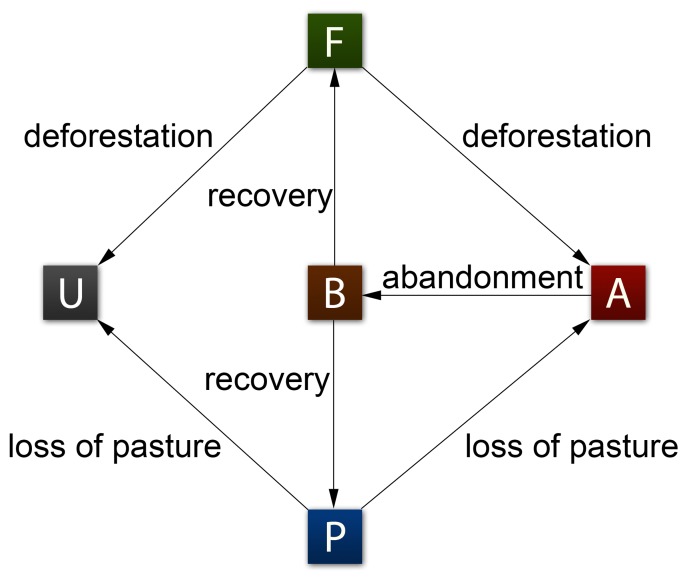
Conceptual depiction of the model. Our model assumes that non-barren land may be in one of five states: forested (F), agricultural (A), wild pasture (P), urban (U) and abandoned (B). The proportions of these land types may change over time in the following ways: forested land and wild pasture may be converted into either agricultural or urban land, agricultural land may become abandoned land, and abandoned land may recover to either forested or agricultural land.

The model is illustrated conceptually in Fig. 1, and the three key inputs to the model are the time-dependent functions of world population, agricultural yield and per capita food consumption. The model captures how transitions between five possible land states–forest, agricultural fields, pasture, abandoned lands, and urban areas– are driven by these three inputs.

Our model is formally written as
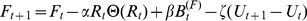
(1)

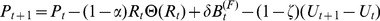
(2)

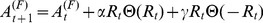
(3)

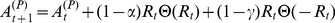
(4)

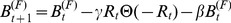
(5)

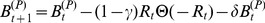
(6)

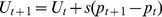
(7)where



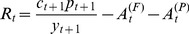
(8)The symbols *F*, *P*, *A* and *U* represent the global area of forested, wild pasture (i.e that not used for agriculture/domestic grazing), agricultural and urban land respectively. The superscripts (*F*) and (*P*) on *A* and *B* are used to keep track of whether the agricultural land was derived from either forested land or pasture. The heaviside functions Θ(•) restrict the directions of land use conversion; for example, agricultural land cannot be converted to forested land without first going through a period of abandonment. The parameters and their values are summarized in Table 1. The model’s processes can be explained in terms of the equations as follows: The *α* and (1−*α*) terms correspond to conversion of forest and pasture (respectively) to agricultural land. The *ζ* and (1−*ζ*) terms correspond to conversion of forest and pasture (respectively) to urban land. The *γ* and (1−*γ*) terms correspond to the abandonment of agricultural land. The *β* and *δ* terms correspond to the conversion of abandoned land to their natural state. The code that was used to generate our results can be downloaded at https://github.com/Pacopag/fpau.

**Table 1:** Summary of model parameters.

Symbol	Description	Value	Source
	Parameters fitted from data		
*T*	Total land area (excluding barren land)	11.26×10^9^ ha	FAO
*α*	Fraction of agricultural land derived from forest	0.4	24
*ζ*	Fraction of urban land derived from forest	0.9	25,26
*s*	Urban area per person	0.06 ha	4
*K_p_*	Maximum un-translated population	10.5×10^9^ people	FAOF
*K_c_*	Maximum un-translated per capita consumption	1940 kg/person/year	FAOF
*K_y_*	Maximum un-translated annual yield	3391 kg/ha/year	FAOF
*r_p_*	Growth rate of population	0.032	FAOF
*r_c_*	Growth rate of per capita consumption	0.019	FAOF
*r_y_*	Growth rate of the annual yield	0.039	FAOF
*p*_0_	Initial population	310×10^6^ people	16
*c*_0_	Initial per capita consumption	571 kg/person/year	FAO
*t_Ip_*	Population inflection time	1998.3 years	FAOF
*t_Ic_*	Per-capita consumption inflection time	1995.8 years	FAOF
*t_Iy_*	Annual yield inflection time	1995.7 years	FAOF
	**Free (fitting) parameters**		
*y*_0_	Initial annual yield	150 kg/ha/year	FP
*γ*	Fraction of abandoned land that is naturally forest	0.4	NS
*β*	Recovery rate of forests	0.01	NS
*δ*	Recovery rate of pastures	1.0	NS

FAO and FAOF indicate data extracted or fitted, respectively, from FAO data. FP indicates a fitting parameter. NS indicates that the model is not sensitive to this parameter in the absence of a forest transition.

Formally, the model contains 19 parameters; however, all but four of these parameters can be fixed using physical and historical data and three of these three are insensitive with respect to model output (Table 1). To achieve a good fit with both FAO and independent data, we tuned only one parameter (i.e. the initial yield, *y*_0_ = 150 kg/ha/year). A detailed description of how the model was calibrated from the available data is given in the following section. The basic processes of our model are similar to those commonly used in models of deforestation and forest transitions [6], [13]. The mechanism of our model is the following: as the population grows and requires more food, agricultural land is created, and as surplus food is produced, agricultural land is abandoned and left to recover to its natural state.

The main driving term in our model is the world food equation; that is, equation (8) which states that agricultural land area *A* is related to population *p*, per capita consumption *c* and agricultural yield *y* by the equation *cp*/*y* − *A* = 0 [7]. Parameters *c*, *p* and *y* all grow logistically to reflect a paradigm shift from a pre-industrialized world to a maximally industrialized one. The mechanistic basis for using a logistic function is the Levins metapopulation model; in a population divided into *N* patches (countries), where each patch can be either “high yield” or “low yield”, and where high yield technology disperses from a “high yield” patch to a “low yield” patch at some rate (thereby converting it to a “high yield” patch), the growth of total population yield is logistic. Since the onset of the Green Revolution such technologies have been transferred to developing countries over time, [14] not unlike metapopulation dynamics, and this transfer explains the majority of global yield gains. A similar argument can be made for per capita consumption trends; as the economies of developing countries grow, their diets tend to become more similar to that of developed countries [15].

The data and justifications we used to fix the parameters and initial conditions are described in detail below. In general, we used historical estimates to fix the initial conditions, and FAO data to fit the growth rates and carrying capacities. The specific details and justifications of the fitting procedure are given in. Logistic behavior of population growth has been well documented, and the world population is expected to stabilize at around 10 billion [16]. Both yield per hectare and consumption per capita have been growing almost linearly over the past few decades. The growth in yield is primarily the result of the Green Revolution of the 1960s [14]. Prior to this revolution yield increases were likely very slow becoming negligible for times before the Agricultural Revolution that took place in Europe in the late 1800s. As we push the limits of technological advancements in the future, we expect yield increases to slow down and eventually stabilize close to a biophysical maximum dictated by energy available in incident sunlight per unit area. Thus, a maximum long-term yield is appropriate. The increase in per capita consumption during the past few decades is greatly attributable to the westernization of the diets people in developing countries [15]. It is reasonable to expect that consumption will also eventually level off as large developing countries complete their economic and dietary transitions. We further assume that urban expansion is simply proportional to population size.

### Model Calibration

**Figure 2:**
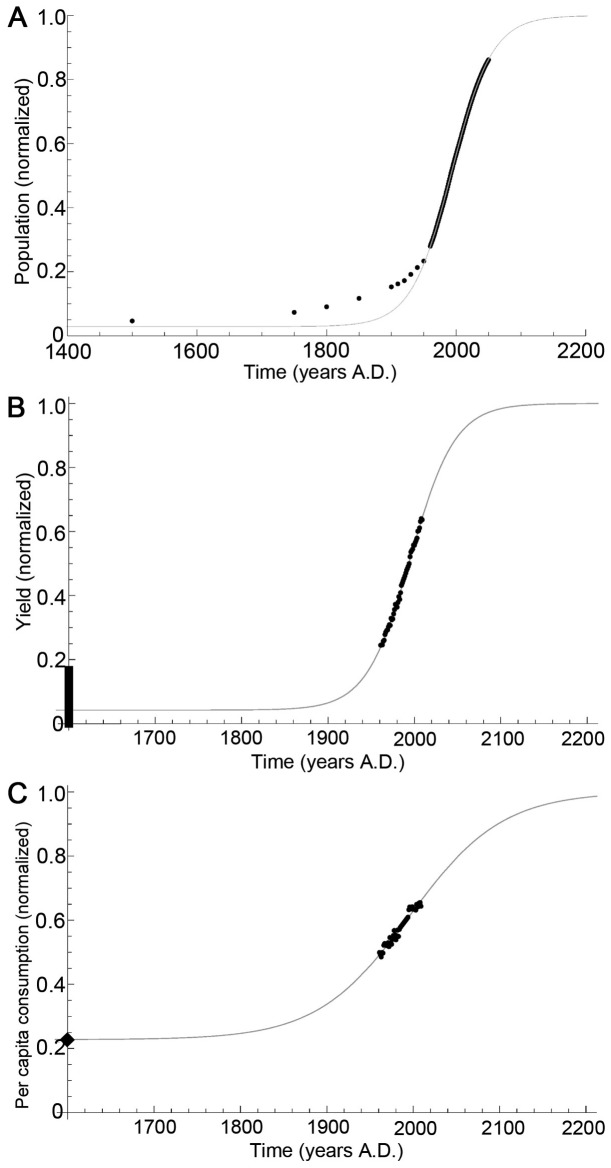
Logistic input curves. (A) Population *p*(*t*)/*p*(∞), (B) yield *y*(t)/*y*(∞), and (C) consumption *c*(*t*)/*c*(∞) as functions of time *t* in years A.D. Black dots represent historical data. Grey curves represent the logistic fit (see eq. (9) and Table 1). In (B), the vertical black line indicates the constraint on the initial yield. In (C), the black diamond represents the per capita consumption in least developed countries in 2009 (FAO). The R^2^ values are 0.992, 0.997 and 0.956 respectively.

The inputs to the model are population *p*, per capita consumption *c* and agricultural yield *y*, which are all time-dependent functions [2], [6]. We model these as logistic functions of the form
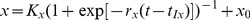
(9)where *x* indicates either *p*, *c* or *y*. The vertical translations of the curves are used to fix the initial conditions. The values of *K_x_* and *r_x_* were fit using a least-squares approach [17]. The parameters and initial conditions are summarized in Table 1. The curves for *p*, *c* and *y* are summarized in Fig. 2.

#### Population

To model the time-dependence of world population *p*, we fitted a logistic curve to data released by the United Nations and the FAO. [16] Our fit is consistent with the UN projection that world population is expected to stabilize in the 23^rd^ century at around 10 billion people due to a demographic fertility transition. There is a discrepancy between the fit and the data between the years 1700 and 1900 because the logistic growth cannot keep up with the anomalous population explosion catalyzed by the industrial revolution. However, this does not affect our conclusions. Urban area was estimated to be at about 3% of the global land area, or roughly 390 Mha, in 2004 [4]. The population was estimated to be 6.43 billion. Together this gives roughly 0.061 hectares of urban area per person, which we use to fix *s*. Although the required urban area per person *s* may actually be time-dependent, we assume that it is changing slowly enough to be regarded as constant over our time scale, especially in comparison to the other input variables *c*, *p*, and *y*.

#### Yield

The yield function *y* is a logistic curve fitted to data extracted from the FAO database. Yield was calculated as total crop, meat and milk production divided by total agricultural land. These data account for both food and non-food agricultural products. FAO data from 1985 to 1993 contain anomalies for some individual fodder crops. These anomalies are not present in the sum of data for all of the agricultural item groups. We removed the anomalous data points and replaced them using linear interpolation. Many models of agricultural yields treat the time-dependence as linear [18], [19]. Indeed, from 1960 until 2009, yields of almost all major crop types and animal products (including our derived average) grew very quickly with a very high degree of linearity. Of course, linear growth cannot be sustained indefinitely, so it is not appropriate for large time scales. Rather, the rapid linear rise in yields that has been observed in recent decades is more likely to level off as humans push the limits of physical bio-energetic constraints on yield through genetic improvements on important crops, and as these technologies are implemented across the globe [2], [14].

Despite some advancements made to farming technology during the middle ages, agricultural yields remained fairly constant until the agricultural revolution, beginning in 17th century Britain, and further accelerated during the industrial revolution [14], [20]-[23]. However, notable yield increases did not make a global impact until the Green Revolution in the middle of the 20th century, when both mechanization and modern varieties of high-yielding crops were beginning to be introduced in many developing countries [14]. The timing of this revolution corresponds very closely with the left endpoint of the available yield data, so the observed linear growth of agricultural yields is largely attributed to the spreading of existing technologies to developing countries, and may not have persisted for very long before the 1960s. Estimates of medieval grain yields suggest that 700 kg/ha was typical of a harvest in those times [20], [22]. Estimates of grain yields in the 19th and 20th centuries suggest a higher, but constant 2000–3000 kg/ha before rapidly increasing in the late 1960s [21], [23]. Less is known about the yield of animal products, but estimates of the number of livestock (equine and bovine) per hectare are given to be in the order of 1 ha^−1^ [21]. However, not all livestock were harvested for food each year, so the annual yield of livestock food products would have been much lower than that for grain (as it is today). From 1960 to 2009, the ratio of the total global yield to that of cereal crops was roughly 0.6. Applying this ratio to estimates of medieval grain yield we constrained the initial yield in our model to be less than 420 kg/ha. On the other hand, the maximum attainable yield of around 3500 kg/ha is determined our fitting algorithm.

#### Consumption

The third driving factor in the world food equation is annual per capita consumption *c*. Global per capita food consumption has been steadily rising over the past five decades. The tendency is for the diets of developing countries to approach that of the United States and other industrialized western countries [15]. We fitted a logistic function to the FAO data for global per capita food consumption (or more accurately, per capita food supply) for the years from 1961 to 2007. As an initial condition we took the value of 571 kg/capita/yr. This value corresponds to the average of per capita consumption in least developed countries, which was found to be almost constant over the period from 1961–1995. The assumption here is that medieval consumption patterns were similar to those presently observed in lesser developed countries. The maximum value c(∞) = 2512 kg/capita/year compares well with the present-day value of about 2300 kg/capita/year for the United Kingdom, which might be expected in a completely industrialized world.

#### Other parameters

In the absence of a forest transition, the model is only sensitive to two external parameters (i.e. not intrinsic to the input function), *α* and *ζ*. We fixed *α* = 0.4 from historical estimates [24]. We did not find any data allowing us to fix *ζ*, but comparing population density maps to biome maps, it is clear that 0<< *ζ* <1 [25], [26]. We coarsely tuned *ζ* to 0.9, but the model is not very sensitive to its variations which can be compensated by small changes in *y*_0_.

#### Initial conditions

To select initial conditions for the state variables *F*_0_, *P*_0_ and *A*_0_^(*F*,*P*)^, we began with a pre- agricultural landscape estimated previously [24]. Then we assumed that agricultural and urban land existed as per the world food equation and equation (8). We took these areas from both forested and pasture land in the proportions dictated by the parameters *α* and ζ.

### Model Fitting Procedure

There are 19 parameters in our model. We were able to fix 15 of these parameters using data and other estimates found in the literature (Table 1) as described in the previous section. For the logistic fits (Fig. 2) we used a least-square method on data translated vertically downward by an amount dictated by the initial conditions, and translated horizontally to center the curve approximately about the vertical axis. Three of the remaining four unspecified parameters (i.e. *γ*, *β* and *δ*) had no effect on the model’s output in the absence of a forest transition. The only remaining unspecified parameter is the initial yield *y*_0_, which is constrained as described in section 2.2. We varied this parameter until we achieved a good fit with both the recent FAO data as well as with estimates of land-type cover in the more distant past.

### Exploring the Model’s Phase Space

In order to probe our model for interesting post-forest transition dynamics, we analyzed the phase diagram of our model using maximum yield *K_y_* and maximum consumption *K_c_* as our control parameters. To do this we smoothly fit new logistic curves to the endpoints of the FAO data on the baseline curves. For any given value of *K_y_* one can fix the value and slope of *y*(*t*) at the enpoint of the data *t_e_ = *2009 by setting
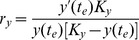
(10)and
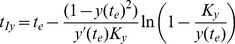
(11)and likewise for 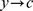 to smoothly fit new logistic curves to the historical ones in Fig. 2, beginning at the endpoint of the FAO data corresponding to the year 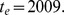 In this way, we scanned across a range of values to compute a section of the phase diagram of the our model.

We also did a sensitivity analysis against all model parameters. The results are summarized in Figure S1. We varied each parameter individually from −10% to 10% of the baseline values while holding all other parameters fixed. We found that the model is quite insensitive with respect to changes in most parameters. In all cases, a relative change of any parameter by 10% resulted in less than 10% a change in land cover fractions. In most cases, the change was much less than 10%.

## Results

### Baseline Scenario: Fitting to Historical Data

**Figure 3:**
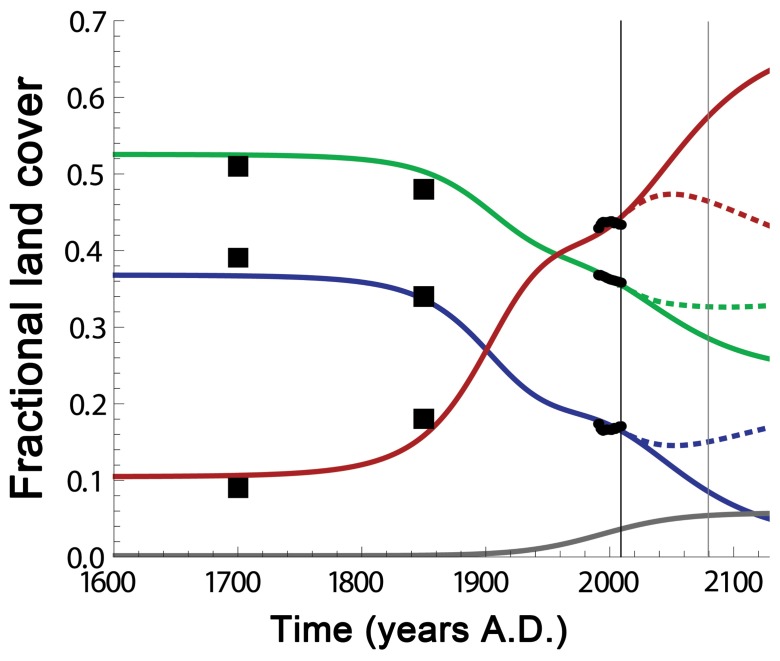
Land cover change over time. Forest area (green), pasture area (blue), agricultural area (red), and urban area (grey) versus time in years A.D. The vertical axis represents the fraction of the total area of non-barren land (1.13×10^10^ ha). The black vertical line corresponds to the year 2009 A.D. The solid black circles represent the FAO data. The solid black squares represent historical estimates. [24] The solid lines correspond to our baseline scenario with the logistic inputs given in Fig. 2. The dashed lines correspond to the case where consumption *c* and yield *y* continue to grow linearly beyond the domain of the FAO data.

Using historical data to fix all but one parameter, the model is able to fit FAO data as well as independent estimates of pre-industrial land cover (Fig. 3) [24]. Projections of land use change for the years 2000 to 2030 have been made in previous studies. For example, 125–416 Mha of new agricultural land, 104–345 Mha of deforestation, and 48–100 Mha of new urban land are expected. [11], [27] These estimates are based on a combination of statistical extrapolation of FAO data as well as by assuming the goals set by the UNFCCC will be met (i.e. to cut the 2010–2020 deforestation rate in half as compared to that from 2000–2010). In comparison, our mathematical model predicts 168, 338 and 132 Mha respectively for new agricultural land, deforested land and new urban land, without the need to assume we can meet UNFCCC goals (Fig. 3). Furthermore, our baseline results for agricultural land cover are in perfect agreement with the “pessimistic” value of 5820 Mha for 2050 derived in an in-depth review of other dynamical models of land use change [10]. Thus, with a few simple processes that are calibrated almost entirely with data, our model simultaneously fits estimates of land use change over several centuries.

Extrapolating far enough in time so that the model’s dynamics stabilize, our baseline scenario (Fig. 3) predicts that a global forest transition is not likely to occur, and that forest and wild pasture cover will stabilize at roughly 25% and 2.5%, respectively, of the non-barren land area. This corresponds to roughly 22% and 1.4% of total land area excluding Antarctica.

### Sustained Linear Growth in Yield and Consumption

Although it is reasonable to expect that both agricultural yield and per capita food consumption will eventually stabilize due to biophysical constraints, we have actually seen almost linear growth in these quantities over the past five decades (Fig. 2). Using a linear extrapolation on these data in our model, we find that a forest transition is likely to occur within the next century if such spectacular growth in yield can be sustained (Fig. 3, dashed lines). In cases in which a forest transition occurs, the model develops sensitivity to the parameter *β*, which determines the rate at which abandoned land becomes re-forested. Although the value of *β* affects the rate at which the forest cover expands, it has little effect on the timing of the forest transition. The reason for this is that the forest transition is driven primarily by the abandonment of agricultural land, which is independent of the re-forestation rate.

### Future Maximal Yield and Consumption Dependence

**Figure 4:**
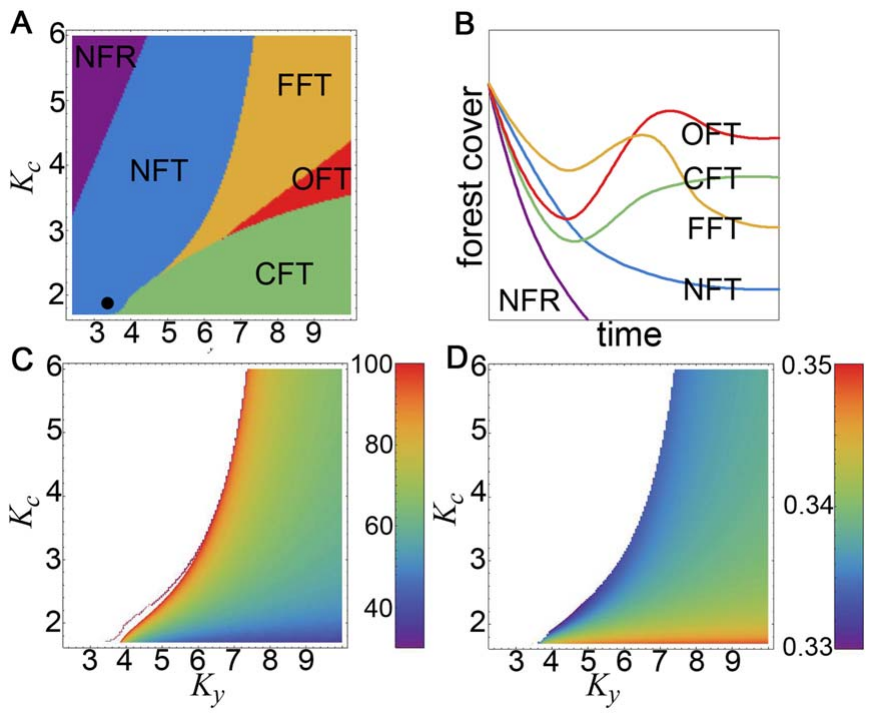
Analysis of *K_y_,K_c_* space. (A) The phase diagram indicates five distinct phases in which forest cover dynamics have distinct qualitative behaviors illustrated in (B): No Forest Remaining (NFR), No Forest Transition (NFT), Classical Forest Transition (CFT), Overshot Forest Transition (OFT), and False Forest Transition (FFT). The solid black circle in (A) corresponds to our baseline scenario. (C) The timing of the forest transition is indicated by the color scale measured in number of years after 2009. (D) The forest cover measured at the time of the forest transition indicated in (C).

The phase diagram (Fig. 4a) shows five distinct phases: No Forest Remaining (NFR), No Forest Transition (NFT), Classical Forest Transition (CFT), Overshot Forest Transition (OFT), and False Forest Transition (FFT). The phases can be understood in terms of the number of turning points in the function *c*(*t*)*p*(*t*)/*y*(*t*). The OFT and FFT (Fig. 4(a) and 4(b)) are examples of non-classical forest transitions. Yield and consumption corresponding to the 2009 values are represented approximately by the axes’ origin. Under the assumption of logistic-like stabilization of yield and consumption, our model predicts that in cases where a global forest transition is likely to occur, it will likely occur within the next century and that the forest cover would be between 33 and 35 percent (Fig. 4(c) and 4(d)). Estimating the scale of reforestation following the transition is encumbered by unknown factors that determine the amount of time for which such increases in yield and consumption will occur. Our model suggests that an ultimate forest cover of less than 40% is typical even of extremely optimistic scenarios.

The phase structure of our model reveals two particularly interesting scenarios in which a forest transition gives way to a second wave of deforestation (Fig. 4 “False Forest Transition”). To our knowledge this is the first model of land use change to capture these dynamics despite strictly increasing population.

**Figure 5:**
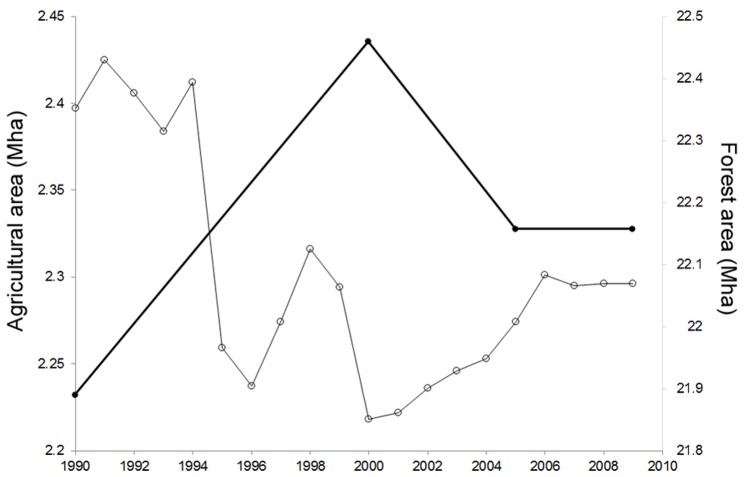
Recent land use dynamics in Finland. The thick line and solid circles represent forest area. The thin line and open circles represent agricultural area. Here we see evidence of a possible “False Forest Transition” where the reforestation following the forest transition (likely to have occurred in Finland’s past) subsequently gives way to a second period of deforestation.

Although our model is a global-level model, the potential for a false forest transition brings into question the sustainability of national-level forest transitions observed in recent decades. For example, we looked at data for land use over the past two decades in Finland and found that the 1990s was a decade in which the forest area expanded significantly in that country, whereas the following decade was marked by a period of subsequent deforestation. Although the scale of deforestation is rather slight, it appears to be coupled with a corresponding decrease of agricultural area in the 1990s followed by an increase in the early 2000s (Fig. 5). Detailed data on forest area for long time scales are rare, so it is currently difficult to do an exhaustive study of the possibility of false forest transitions in other countries.

### Agricultural Land-sparing Strategies

Next we considered the question as to which strategy would spare the most land: increasing yield, or inhibiting increases in consumption. We found that independently increasing *K_y_* or decreasing *K_c_* from the baseline value indicated in Fig. 2 by a given percentage had roughly the same effect on the ultimate forest cover. Yield increases depend on the continued success of genetic improvements of crops and are ultimately limited by biophysical constraints, and much of the recent increase in yield has occurred through transmission of existing technologies to developing countries, not necessarily through development of new technologies [14]. On the other hand, inhibiting consumption increases may seem to require a change in the global conscience about food intake, but there are other more controllable ways to spare land through reduced consumption. For example, an estimated 32% of all food is lost or wasted every year [27]. If this could be reduced to just 29%, then our model predicts that almost 230 Mha of land would ultimately be spared with respect to our baseline scenario in Fig. 3. This is in good agreement with estimates of land sparing for similar reductions in food wastage by 2030 [27]. Moreover, a 5% decrease in per capita food intake could spare as much as 158 Mha of forest and wild pasture.

Although the situation for wild pastures appears rather dire in our baseline scenario, it should be noted that the FAO reports a considerable amount of wild pasture as agricultural land. So the 2% pasture cover should be interpreted as pasture that is untouched by domestic grazing livestock. Over the past 50 years, a fairly constant 69% of agricultural land was categorized as permanent pasture. Thus, the actual pasture cover (wild and cultivated) is more like 47%. This implies that human agricultural activities will have *created* pasture in addition to the natural global pasture cover, although very little of it will be completely natural. It should also be mentioned that agricultural land includes crops coming from trees (e.g. fruits and nuts), which could add to the global forest cover. However, these crops constitute less than 2% of all of the agricultural area harvested from 1961–2009, so the significance is small. On the other hand, the area occupied by these crops has more than doubled over this period. If this trend continues, land area covered by tree crops may account for a significant portion of the global forest cover in the future.

## Discussion

Here, we showed that a mathematical model based on the world food equation explains past land use patterns at the global scale, and over the past millennium. The model requires only one free parameter. The same model predicts that a global-level forest transition is unlikely at baseline parameter values, and would require very significant changes in technology and/or consumption patterns. While this is not the first time these predictions have been made, mathematical models of this type are underutilized at the global level, and the model has shown how our lexicon regarding the future of forest cover must expand beyond the simple forest transition/no forest transition dichotomy to include possibilities such as false forest transitions and overshot forest transitions (Figure 4).

It should be noted that our model implicitly assumes a “business as usual” scenario as it extrapolates on contemporary trends in consumption and yield. While efforts to improve agricultural yield will likely continue, there are also possibilities for increased widespread demand for food that is not particularly land-intensive. For example, increased dependence on aquaculture and insect protein could have an enormous impact on reducing pressure on the world’s remaining natural land habitats. There are other technologies that we do not consider here that could potentially increase yield to a level that would almost certainly induce a forest transition. For example, *in vitro*-cultured meat or the widespread adoption of alternative protein sources could change the face of the planet [28].

Our predictions rely on the assumption that the inputs to the world food equation, *p*, *c* and *y*, will all stabilize in a future where the world has become maximally industrialized. If this will be the case, and we are already observing national-level forest transitions in industrialized countries across the world, then we should consider the reasons why a global-level forest transition may not occur. A national-scale forest transition is often heralded as a great success in forest management and conservation; however, some countries achieve this simply by importing more food and forest products [11]. By evaluating dynamics at the global level, we avoid this confounding factor. Our model finds that a forest transition occurs when yields do not increase fast enough to keep up with growing consumption. In this light, a forest transition may be viewed as a mismanagement of forest and agricultural resources, or a crisis in population growth until a certain point in time when policy, increasing yields, imports, or other factors halt further deforestation [11]. In light of the suggestion that regenerating forests may have lesser or different ecological quality than the original forest, [12] perhaps the best-case scenario is not a global forest transition, but for forest cover to settle at its natural equilibrium corresponding to an ultimate stable population.

Some researchers have suggested that the factors *p*, *c* and *y* may not have the effects on deforestation and land use that might be expected by conventional wisdom. For example, the inverse relationship between population and deforestation has been shown to be weakening in some countries in recent decades, which is likely due to increasing yields. [1] Yield gains on commodities that have elastic demand may actually promote agricultural expansion and thus deforestation [1], [29]. We account for these effects with time varying consumption and yield terms. Also, local decreases in yield may lead to agricultural abandonment and decreased pressure on local forests, but so-called “leakage” effects simply transfer the pressure to other localities, thereby resulting in net deforestation [7], [11]. Here, in contrast, we avoid such confounding factors by focusing on global-scale land use changes and the prospects of a global forest transition.

Efforts to conserve forest ecosystems are often directed at setting aside tracts of land in countries that can import whatever they need. However, the long-term explanatory power of the world food equation as we have demonstrated here, together with the observation that many national-level forest transitions may essentially be luxury imports, suggest that equal effort should be directed toward finding ways to boost agricultural yield, disseminate those technologies to developing countries, and decrease per capita consumption, thus reducing land use pressures [7].

## Supporting Information

Figure S1 **Sensitivity analysis.** The horizontal axis is the percentage change of the corresponding parameter from its baseline value. The vertical axis is the absolute change in the fractional land cover. Varying the excluded parameters has a negligible effect.
(DOCX)
