# Supplementary material for: Outlook on a Worldwide Forest Transition
Source: PLoS One. 2013 Oct 9;8(10):e75890. doi: 10.1371/journal.pone.0075890 (PMC3794021; doi:10.1371/journal.pone.0075890)
Supplement: Figure S1 — Sensitivity analysis. The horizontal axis is the percentage change of the corresponding parameter from its baseline value. The vertical axis is the absolute change in the fractional land cover. Varying the excluded parameters has a negligible effect. (DOCX) [file pone.0075890.s001.docx]

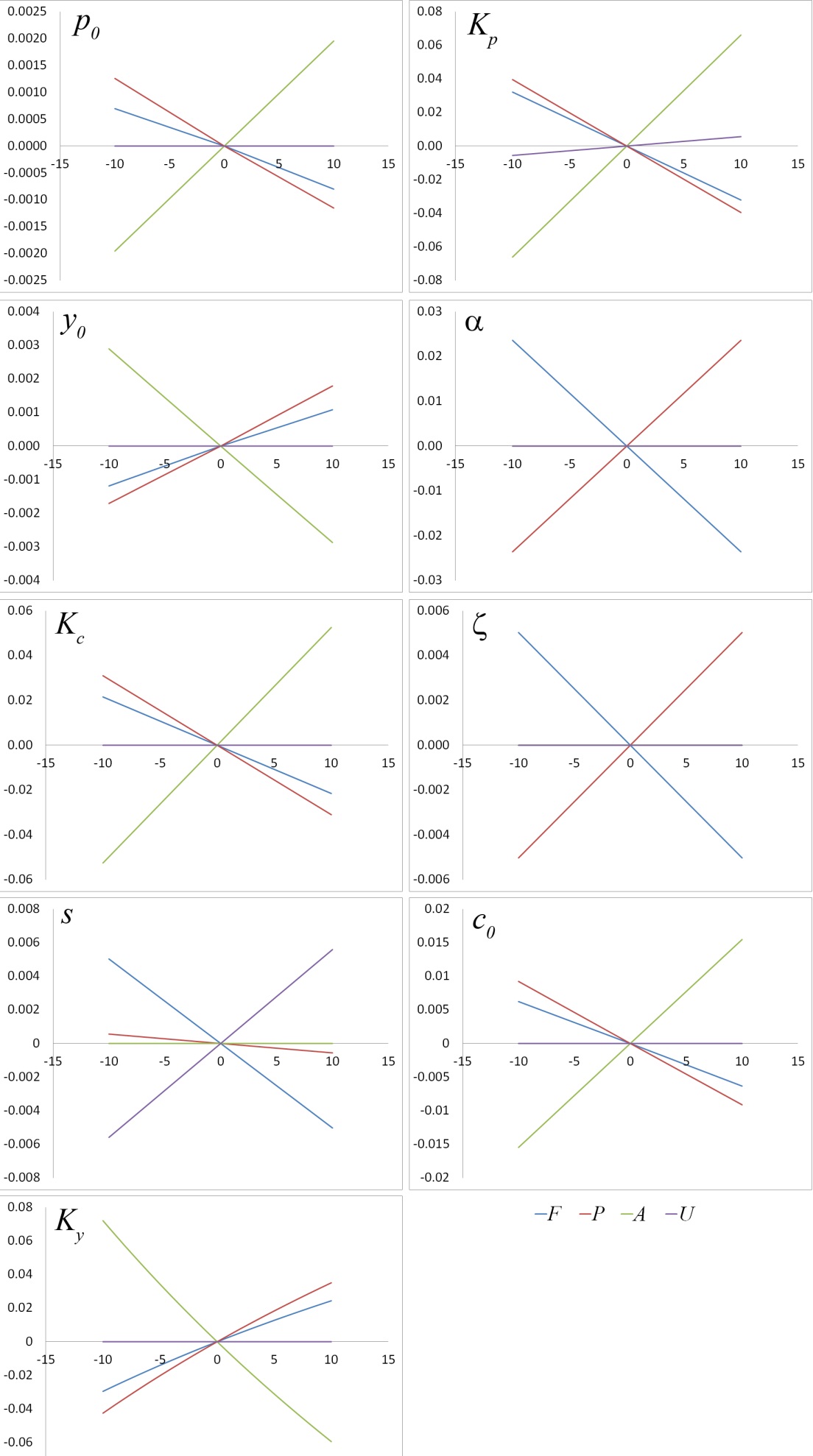


Supplementary Figure S1 – Sensitivity analysis. The horizontal axis is the percentage change of the corresponding parameter from its baseline value. The vertical axis is the absolute change in the fractional land cover. Varying the excluded parameters has a negligible effect.
